# A Catastrophic Cascade of Postoperative Complications Following Hemiarthroplasty for Femoral Neck Fracture in a Middle-Aged Patient With Schizophrenia

**DOI:** 10.7759/cureus.9044

**Published:** 2020-07-07

**Authors:** Eustathios Kenanidis, Sofia-Chrysovalantou C Zagalioti, Nikolaos Milonakis, Evangelia M Tsapakis, Eleftherios Tsiridis

**Affiliations:** 1 Academic Orthopaedic Department, Papageorgiou General Hospital, Aristotle University Medical School, Thessaloniki, GRC; 2 Center of Orthopaedic and Regenerative Medicine - Center of Interdisciplinary Research and Innovation, Aristotle University Medical School, Thessaloniki, GRC; 3 1st Academic Department of Psychiatry, Papageorgiou General Hospital, Aristotle University Medical School, Thessaloniki, GRC; 4 Psychiatry, Agios Charalambos Mental Health Clinic, Heraklion, GRC

**Keywords:** hip fracture, sub capital hip fracture, schizophrenia, hemiarthroplasty, dual mobility cup, constrained liner, girdlestone procedure, postoperative complications, femoral neck fracture

## Abstract

The treatment of a patient with schizophrenia suffering a subcapital hip fracture may be challenging, mainly due to the high risk of postoperative medical and surgical complications. Mechanical complications from the implant are also frequently encountered following hip arthroplasty in patients with schizophrenia. We report the case of a 57-year-old male patient with schizophrenia who underwent hip hemiarthroplasty for a displaced femoral neck fracture. During the initial postoperative period, the patient developed a cascade of surgical and mechanical complications, leading to multiple revision procedures and a suboptimal outcome. The ideal type of treatment of patients with schizophrenia with subcapital hip fracture is still missing. It is, therefore, important to highlight the high risk of postoperative complications in patients with schizophrenia who present with subcapital fractures subsequently treated with hemiarthroplasty.

## Introduction

Schizophrenia is a chronic, severe mental illness characterized by relapsing episodes of psychosis, during which reality is interpreted in an abnormal way [1]. Hallucinations, delusions, disorganized thinking, and social withdrawal are the major symptoms of this chronic and severe mental health illness leading to considerable disability. Up to 0.7% of people will be affected by schizophrenia during their lifetimes due to environmental and genetic factors and the majority are young males [2]. People with schizophrenia are two times more likely to die earlier than the general population as they often suffer from preventable physical diseases, such as cardiovascular disease, metabolic disease and infections [3]. Moreover, complex psychopharmacologic treatment, including high doses of antipsychotics, seems to predict a lower hip bone mineral density and a higher risk of sustaining hip fractures in patients with schizophrenia compared to gender-matched healthy controls [4-7]. Surgical repair is the treatment of choice for hip fractures, but the type of treatment depends on the patient’s age, his or her pre-injury level of function, and the type and displacement of the fracture [8]. Open reduction and internal fixation (ORIF), hemiarthroplasty and total hip arthroplasty (THA) are the main treatment options for femoral neck fractures [8]. The type of treatment for psychiatric patients with hip fractures has, however, not been extensively evaluated to date.

Surgical patients with schizophrenia are reported to be at a higher risk of postoperative pulmonary, renal, neurological and blood vessel complications, in addition to 30-day mortality, compared to patients without psychiatric illness [9]. Furthermore, following trauma and elective orthopaedic procedures, patients with schizophrenia demonstrated a greater risk of postoperative mechanical implant and surgical complications with longer hospital stays compared to patients with other psychiatric comorbidities or healthy controls [10,11].

## Case presentation

We here report the case of a 57-year-old male patient with schizophrenia who suffered a displaced femoral neck fracture and was initially treated with hip hemiarthroplasty. During a short follow-up period, the patient developed a cascade of postoperative surgical and mechanical complications. As a result, he underwent multiple revision procedures that resulted in a suboptimal outcome.

**Figure 1:**
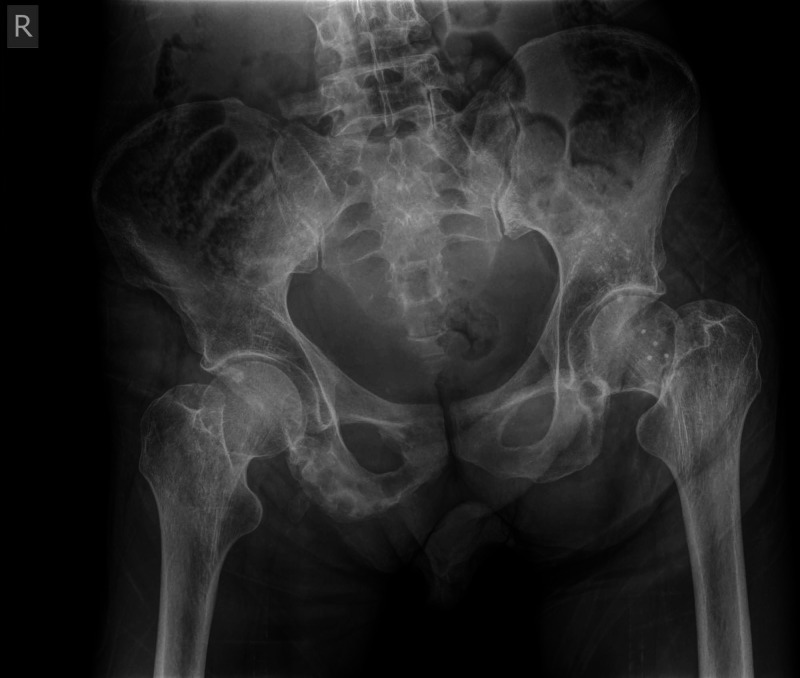
Preoperative anteroposterior pelvis X-ray demonstrating the subcapital fracture of the left hip

A 57-year-old male with a history of schizophrenia sustained a displaced left femoral neck fracture after a fall (Figure 1).

The patient was notably sarcopenic and previously independently ambulating. He was accommodated in a psychiatric institution for years. He was under chronic antipsychotic treatment with zuclopenthixol, biperiden, chlorpromazine and haloperidol, but had no further comorbidities nor previous surgeries. On admission to our orthopaedic department, the patient was neurovascularly intact distally.

**Figure 2:**
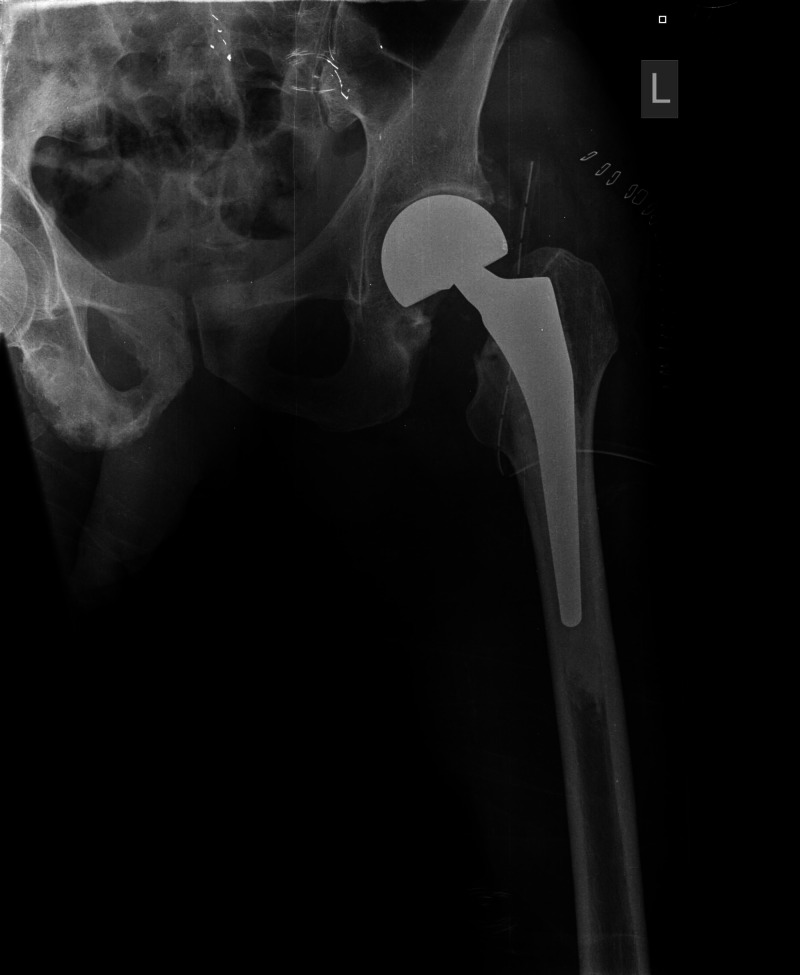
Postoperative anteroposterior radiograph of the left hip showing the left hip cemented bipolar hemiarthroplasty

The patient underwent a left hip cemented bipolar hemiarthroplasty through a posterior approach (Figure 2).

Intraoperatively, a substantial gluteal and short external rotator muscles atrophy was recognized. No adverse perioperative events were reported. The wound was healing well, and the patient was encouraged to ambulate with partial weight-bearing for 30 days. Postoperatively, the patient used to hold his left leg in flexion and adduction persistently. The patient was discharged from the hospital under low molecular weight heparin medication and instructions to maintain hip precautions for a month.

**Figure 3:**
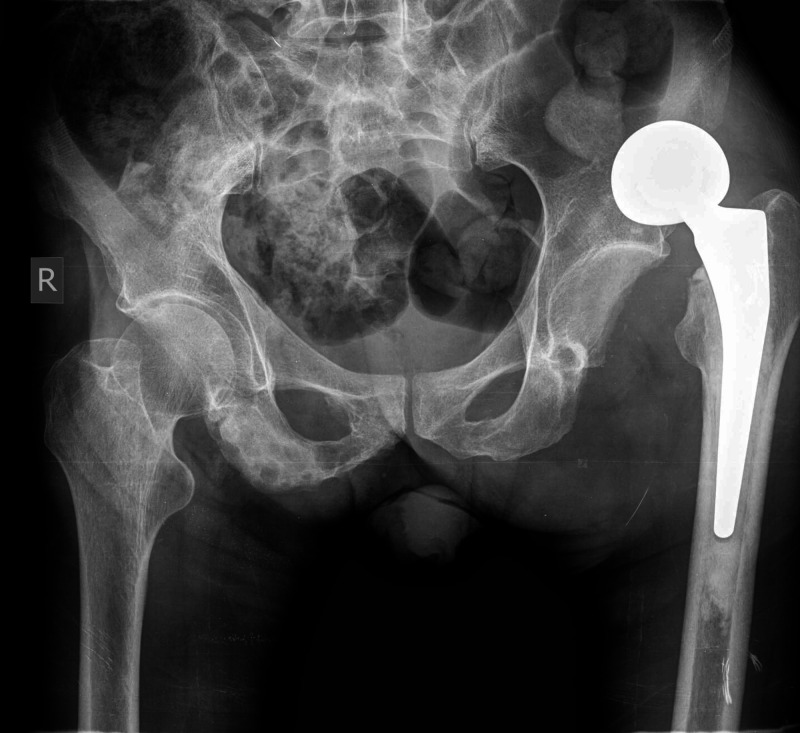
Postoperative anteroposterior pelvis radiograph demonstrating dislocation of bipolar hemiarthroplasty of the left hip

Forty days postoperatively, the patient was readmitted to our orthopaedic department complaining of acute left hip pain and inability to bear weight. No previous fall was reported. Radiographic evaluation revealed a dislocation of the left hip hemiarthroplasty (Figure 3).

**Figure 4:**
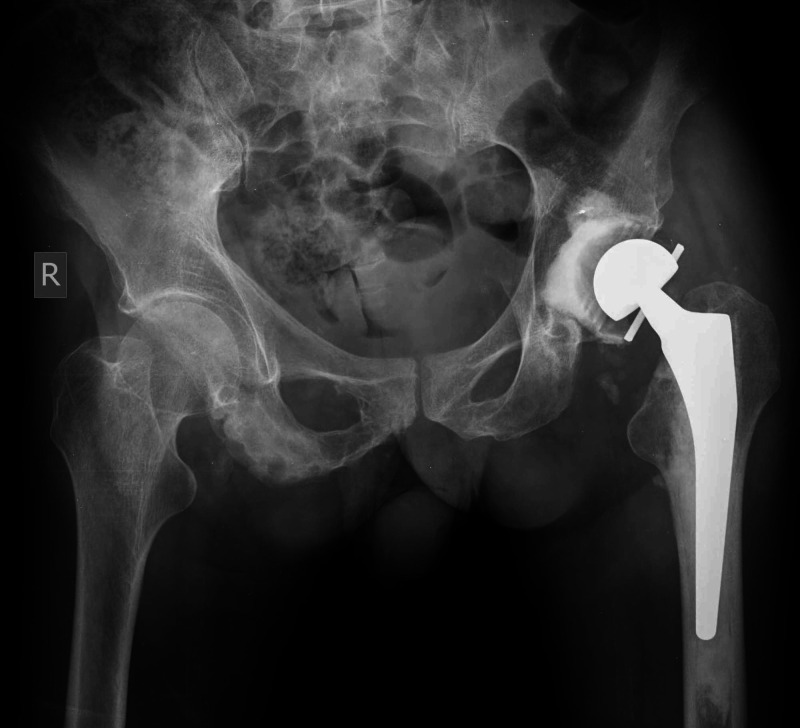
Postoperative anteroposterior pelvis radiograph showing revision of hemiarthroplasty of the left hip to a total hip arthroplasty with cemented constrained liner

Revision hip arthroplasty was then performed through the initial approach. The hemiarthroplasty was revised to a THA with cemented constrained liner; the femoral stem was deemed stable and was not revised (Figure 4).

The immediate postoperative period was uneventful. The patient was mobilized with partial weight-bearing and eventually discharged.

**Figure 5:**
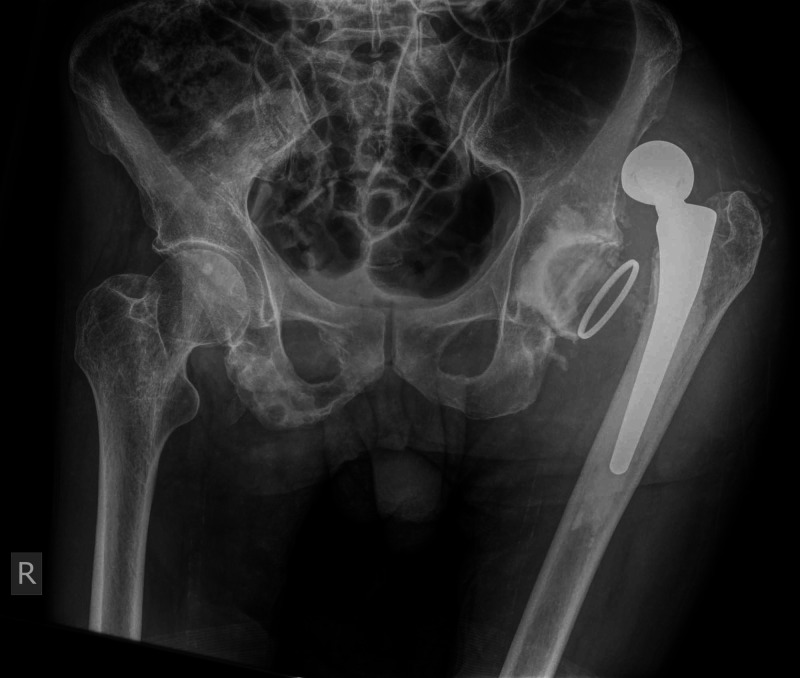
Postoperative anteroposterior pelvis radiograph demonstrating dislocation of cemented constrained liner of the left total hip arthroplasty

Forty-four days later, however, the patient was readmitted to our department with a dislocated constrained left THA (Figure 5).

**Figure 6:**
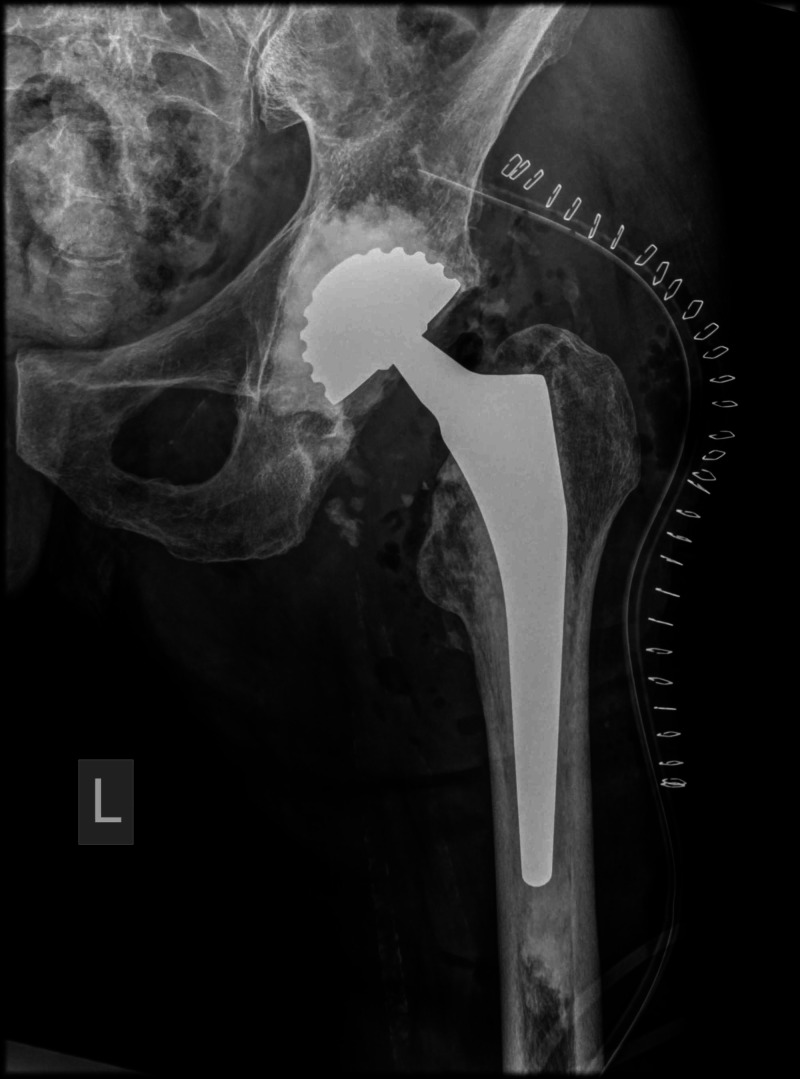
Postoperative anteroposterior left hip radiograph demonstrating re-revision of cemented constrained liner to a cemented dual-mobility cup of the left total hip arthroplasty

The cemented constrained liner was then re-revised to a cemented dual mobility cup (Figure 6).

The femoral stem was preserved and the adductors and iliopsoas tendons were released. Postoperatively, all the precaution measures were taken to prevent further dislocation. 

**Figure 7:**
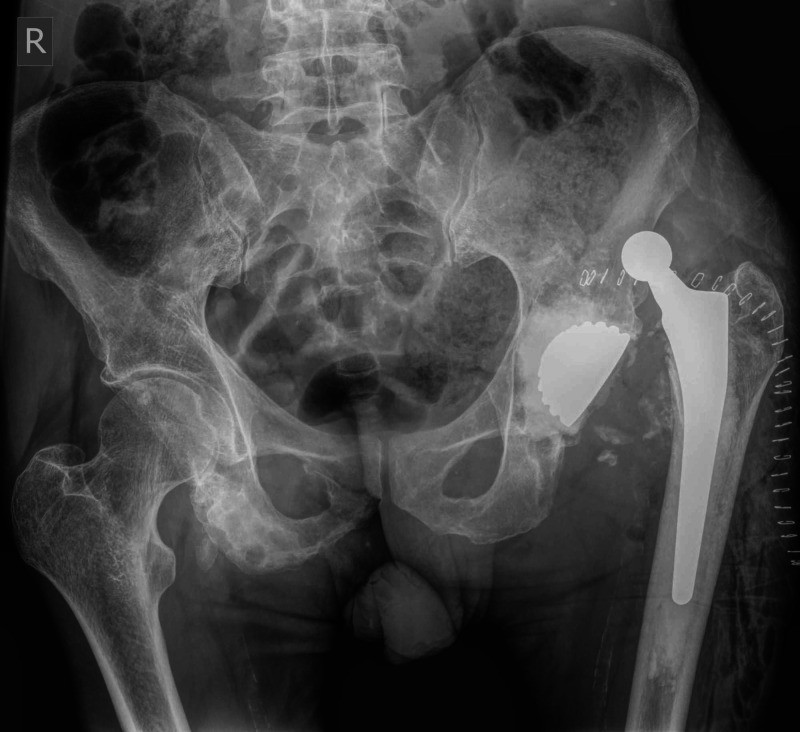
Postoperative anteroposterior pelvis radiograph demonstrating dislocation of a cemented dual-mobility cup of the left total hip arthroplasty

However, during the following month of his hospital stay, he sustained a dislocation of the dual mobility hip (Figure 7).

**Figure 8:**
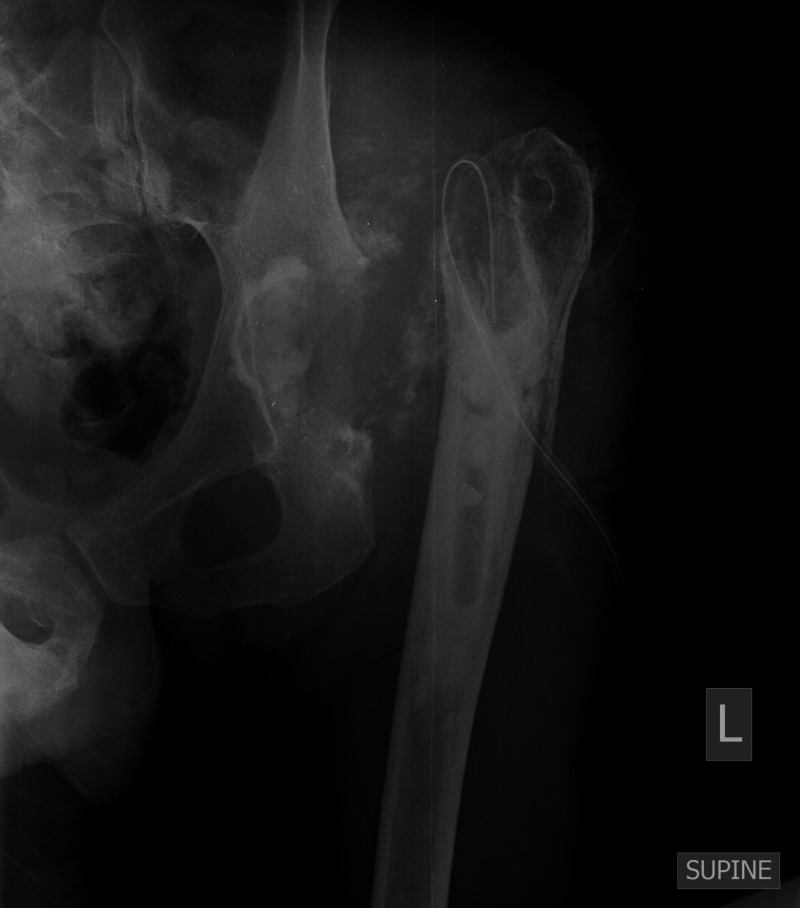
Postoperative anteroposterior left hip radiograph showing the resection Girdlestone procedure, removal of all implants and the acetabular cement

Moreover, the patient became refractory to antipsychotic medication. The surgical wound deteriorated, and eventually wound dehiscence occurred. Pseudomonas aeruginosa was isolated from the wound and treated accordingly. Τhe dislocation was successfully managed with closed reduction under sedation. However, due to the continuous wound dehiscence, a resection Girdlestone procedure was decided (Figure 8).

All implants and acetabular cement were removed. Thorough wound debridement with removal of necrotizing tissues and irrigation with pulsed lavage was performed. Acinetobacter baumani and proteus mirabilis were identified from cultures obtained intraoperatively, and antibiotic treatment was readjusted accordingly.

**Figure 9:**
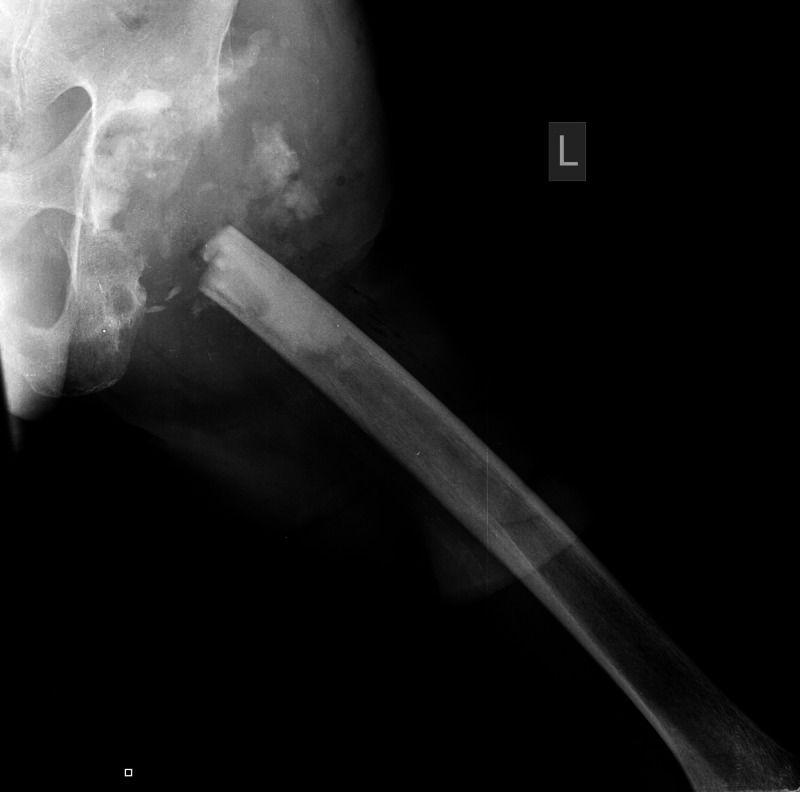
Postoperative anteroposterior left hip radiograph showing the radiologic outcome of the last procedure where the proximal femur was osteotomized to safe margins

Wound healing was delayed, however, and the underlying proximal femur was gradually exposed through the skin wound. As the patient was not cooperating, an MRI could not be performed to evaluate the extent of osteomyelitis. Further surgical wound debridement and a distal femoral osteotomy to healthy margins were subsequently performed (Figure 9).

Antipsychotic medication was also revised to include antidepressants and atypical antipsychotics in order to improve mental health symptoms. Hypoalbuminemia was actively corrected, to control infection. Eventually, the surgical wound healed well, and all laboratory tests returned to normal. Resolution of local and systematic infection contributed to stabilization of the patient’s mental state. The patient was discharged back to a long-term mental health facility, being able to sit, but he could not weight-bear on the left side, as expected.

## Discussion

Chronic mental illness has been recognized as a risk factor for postoperative complications following trauma and elective orthopaedic surgery [11-13]. Patients suffering from major depression, bipolar disorder and schizophrenia are at a higher risk for medical complications, periprosthetic infections, fractures and dislocations during the first 90 postoperative days after THA [13]. Poor socioeconomic and health status, difficulties in communication, interaction of antipsychotics with perioperative medications are believed to contribute to the higher risk of complications in mentally ill patients [11-13].

Schizophrenia is related to higher than expected risk for postoperative complications. Moreover, in patients undergoing surgery for femoral neck fractures, schizophrenia has been recognized as an independent risk factor for any adverse event following surgery, including acute postoperative mechanical complications and prolonged hospital stay [11]. Among 68,000 patients suffering from femoral neck fractures treated with hemiarthroplasty, patients with schizophrenia demonstrated higher, almost double the risk for revision surgery [12]. In our case, the cascade of postoperative complications presented above highlights the increased risk for adverse events after hemiarthroplasty for hip fractures in patients with schizophrenia. This is believed to be secondary to poor patient compliance with weight-bearing, poor co-operation with physiotherapy and failure to obey instructions for movement restrictions.

The age of the patient, his pre-injury level of function and the displacement of the fracture usually determine the type of surgical treatment for subcapital fractures [8]. Optimally, in young patients, sliding hip screws or cannulated cancellous screws are used to internally fix displaced femoral neck fractures, with no confirmed superiority of the one over the other [14]. Arthroplasty is the primary surgical choice in elderly patients with displaced femoral neck fractures in order to achieve early mobilization. However, there is no consensus on whether hemiarthroplasty or THA should be preferred [15,16]. Similar clinical outcomes have been reported for THA and ORIF in the treatment of middle-aged patients with subcapital fractures; THA is more cost-effective over the age of 50, depending on patients’ comorbidities [17].

It remains unclear which is the best type of surgical treatment for patients with schizophrenia who undergo subcapital hip fractures. Here, the initial decision to treat our patient with hemiarthroplasty was based on his biological age, his chronic and severe mental illness and the institution he was living in and to which he would be discharged to. Furthermore, in cognitively impaired and high-risk patients, constrained or dual-mobility liners are used for the prevention of dislocation, but data in patients with schizophrenia remains inconclusive [18,19]. On the other hand, ORIF has not been evaluated in patients with schizophrenia. In our case, postoperative complications may have been avoided, had we had internally fixed our patient with a sliding hip screw, in the first place. Besides, hemiarthroplasty through the anterior or the lateral approach might be preferable in patients at higher risk for dislocation [20].

Preoperative optimization of the psychiatric patient is crucial for achieving the best possible outcome. In order to avoid postoperative complications, improve patient compliance and maximize the surgical outcomes in patients with schizophrenia, a thorough preoperative multidisciplinary patient care plan should be drafted and implemented. Close co-operation between psychiatric and orthopaedic surgical teams seems to be of paramount importance. Continuous nursing observation to ensure compliance with mobility restrictive measures in addition to regular specialized physical therapy should be part of the postoperative treatment plan and are strongly recommended.

## Conclusions

Schizophrenia is an independent risk factor for postoperative complications following the surgical management of hip fractures. Medical, surgical, and mechanical complications are common after hemiarthroplasty for displaced femoral neck fractures in patients with schizophrenia. Co-operation between orthopaedic surgeons and psychiatrists, physiotherapists, psychologists, nursing staff, occupational therapists and social workers, is the cornerstone of a successful outcome. Optimal surgical treatment option remains to be determined; however, as THA and hemiarthroplasty show a high risk of dislocations for the management of femoral neck fractures in patients with schizophrenia. Dual-mobility and constrained liners in THA, and cannulated or sliding hip screws have not been extensively evaluated. Further research is needed to develop more detailed management algorithms for these challenging cases.
